# The Chap-Book

**Published:** 1895-02

**Authors:** 


### BOOK NOTICES.

THE CHAP-BOOK; semi-monthly; published by Stone & Kimball, Caxton Building, Chicago, Ill. Subscription price, $1 per year; five cents a number.

This is a quaint periodical of about fifty pages, small octavo. Its quaintness is much increased by the old-style type, unsized paper, and rough edges.

As its name is somewhat uncommon, we turn to *The Standard Dictionary* of Funk & Wagnalls, and copy therefrom the definition:

"CHAP-BOOK: One of the cheap books usually in pamphlet form, once popular in England, Scotland, and the American Colonies, containing tales, ballads, lives, tracts, etc.: sold by chapmen."

The December number, now before us, contains a variety of interesting material. One article is so weird that we quote scraps from it. It is too long to reproduce in full.

THE NIGHT WASHERS.

"Whe—ooh, ooh, ooh, ooh, ooh,
We are the brothers of ghouls, and who
In the name of the Crooked Saints are you?

"We are the washers of shrouds wherein
The lovers of beauty who sainted sin
Sleep till the Judgment Day begin.

"When the moon is drifting overhead,
We wash the linen of the dead,
Stained with yellow and stiff with red.

"Whe—ooh, ooh, ooh, ooh, ooh,
We are the foul night washers, and who
By the seven lovely sins are you?"

There are some illustrations in the book all in a style as weird as is the sample of text above quoted. They are by Will H. Bradley, and are the most original and striking designs we have ever seen. They are masses of black and white, with long, sweeping curves, quite bewildering. It is a new type of illustration and very singular.

